# A first-year maize/cassava relay intercropping system improves soil nutrients and changes the soil microbial community in the symbiotic period

**DOI:** 10.3389/fmicb.2023.1087202

**Published:** 2023-03-21

**Authors:** Cuicui He, Bin Zhou, Hua Wang, Yunxia Wei, Jie Huang

**Affiliations:** ^1^Tropical Crops Genetic Resources Institute, Chinese Academy of Tropical Agricultural Sciences, Haikou Hainan, China; ^2^Eco-environmental Protection Research Institute, Shanghai Academy of Agricultural Sciences/Shanghai Scientific Observation and Experimental Station for Agricultural Environment and Land Conservation/Shanghai Environmental Protection Monitoring Station of Agriculture/Shanghai Engineering Research Centre of Low-carbon Agriculture (SERLA)/Shanghai Key Laboratory of Protected Horticultural Technology/Key Laboratory of Low-carbon Green Agriculture in Southeastern China, Ministry of Agriculture and Rural Affairs, Shanghai, China

**Keywords:** relay intercropping system, maize/cassava, soil nutrients, microbial community, rhizosphere/non-rhizosphere

## Abstract

The maize/cassava relay intercropping system can be a specific and efficient cropping pattern in a tropical/subtropical field. Relay intercropping systems contribute to the optimization of land use, fostering sustainable and efficient agriculture. This study entails a first-year comparative intercropping experiment using maize (*Zea mays* L.) and cassava (*Manihot esculenta* Crantz). We determined the soil nutrient contents and physicochemical properties as well as the microbial communities by high-throughput sequencing. We found that the relay intercropping system changed the bacterial community structure, especially the rhizosphere microorganisms. The bacterial community was characterized by a higher abundance of the phyla Chloroflexi, Proteobacteria, and Actinobacteria and of the genus *AD3*, which are related to nutrient cycling and decreased bioavailability of Cd. At the same time, the relay intercropping system reduces the relative abundance of pathogenic bacteria and biomarkers of organic pollution. The relay intercropping systems significantly boosted soil pH and available K levels in addition to the monocropping yield. The results of this study suggest that maize/cassava relay intercropping, starting from the first year, not only provides a stable supply of food and income due to the diversified cropping systems but is also beneficial for the improvement of soil quality and the soil microbial community.

## 1. Introduction

In view of the rapid growth of the global population, which is expected to reach 10 billion people by 2050, and of the resulting shortage of natural resources, there is an urgent need to improve food yield (Caira and Ferranti, 2016). Although intensive agriculture can increase crop yields by increasing nitrogen fertilizer inputs, the efficiency of nitrogen fertilizer use is reduced, resulting in nitrogen waste and subsequent environmental problems (Wang et al., 2014; Morrissy et al., 2021). As one of the most populous and developing countries, China encompasses 7% of the world’s farmland but feeds nearly 20% of the world’s population (Larson, 2013). Faced with such severe challenges, finding a planting model that can effectively increase yield and improve nutrient use efficiency has become an urgent and important research topic. Intercropping is the practice of growing two or more crops on the same plot of land during the same growing season (Mao et al., 2015). This practice is widely recognized for maintaining and improving soil quality and subsequent crop yields (Chen et al., 2019; Cuartero et al., 2022).

Intercropping has several advantages, including efficient nutrient acquisition, reduced soil-borne diseases, reduced use of herbicides and pesticides, and increased microbial diversity in the farmland soil–plant system (Mousavi and Eskandari, 2011). Mechanically combining different crops does not necessarily increase yield in an intercropping system, but rather effective yield improvement depends on the balance between crops in an intercropping system (Hailu, 2015). The main intercropping systems widely used in the world are strip and relay intercropping systems. Relay intercropping is different from intercropping in the coexistence period, and there are differences in planting time and space (Lichtfouse, 2009). Effective yield improvement depends on the balance between crops in the intercropping system, and the relationship of the intercropping system is not clear.

Maize has received attention in intercropping systems with legumes, pepper, peanut, alfalfa and other crops because of its positive effect on crop yield and nutrient use efficiency (Gao et al., 2021; Jiao et al., 2021; Zhang et al., 2021; Ben-chuan et al., 2022; Wu et al., 2022). Cassava, a widely grown crop in the tropics and subtropics, is relatively drought-tolerant and highly resilient to climate change and is regarded as an important crop for food security (Enesi et al., 2022). The substantial potential of maize/cassava relay intercropping systems to increase yield and improve soil nutrient status has been widely reported (Okorie et al., 2020; Wahab et al., 2022). The impact of intercropping systems on soil nutrients and soil microorganisms is extremely significant (Gao et al., 2021; Ben-chuan et al., 2022; Cuartero et al., 2022). Crops use nutrients and space differently in space and time, so combining them in the right way is particularly important for crop yield improvements. To the best of our knowledge, the study of maize and cassava intercropping systems and the intercropping patterns of these two crops with regard to soil nutrient changes is still incipient, and there are few reports on the functions and responses of soil microorganisms to intercropping. However, this combination can be an important option for sustainable horticultural management. For long-term crops, such as cassava, growth and leaf area development per unit of thermal time slowly increase in the initial stage (Silva et al., 2016). Maize, as a long-lasting crop, matures within 3–4 months, thus offering opportunities for enhanced radiation capture and possibly reduced soil moisture loss (Nwokoro et al., 2022). Cassava and maize intercropping adopts maize and cassava planting at the same time; when the maize is harvested, cassava is still at the seedling stage. Intercropping had some effect on the cassava seedling stage, but this effect was small compared to the increased light and heat captured by the maize, increased yields and reduced weed damage (Nwokoro et al., 2022). Previous studies have mainly focused on the effects of total soil nutrients and fertilization and have not linked the changes in microbial diversity caused by the implementation of intercropping with the availability of nutrients. Moreover, studies on relay intercropping considering the effects of time and space are also limited. Therefore, we chose the rhizosphere and non-rhizosphere soils in the symbiotic period of a one-year relay intercropping system as the research object to study the changes in soil nutrients and chemical properties, as well as in soil bacterial diversity and community composition, and to determine the relationship between these changes and soil chemical properties and crop yield compared to monoculture systems.

## 2. Materials and methods

### 2.1. Experimental design and sampling

Experiments were conducted from 2020 to 2021 at an experimental farm in Guiping, Guangxi Province, China (23°19′43″N, E109°53′39″E). The climate of the experimental site was subtropical monsoon. The main soil type is loam red soil, and the farming system is two crops a year. In dry land, the main crop is cassava, intercropping peanut, corn and other crops. The local farm area is approximately 100 hectares. The experiments were conducted in soil that had been cultivated with cassava and maize patterns for more than 10 years. Before the experiment, the soil had a pH of 4.10, 35.6 g kg^−1^ organic matter, 1.11 g kg^−1^ total N, 96.6 mg kg^−1^ available N, 217 mg kg^−1^ available P, and 46.0 mg kg^−1^ available K in the 0–20 cm soil layer.

The maize/cassava relay intercropping system was used in the field experiments. The three treatments with three replications were as follows: (1) sole cassava: the intrarow and interrow spacing of cassava plants was 80 and 100 cm, respectively. (2) Cassava and maize intercrops: the intercropping patterns utilized two rows of maize and one row of cassava. The intrarow and interrow spacing of maize plants was 40 and 50 cm, respectively. While cassava was planted at the center point of four maize plants, the intrarow and interrow spacing of cassava plants was 80 and 100 cm, respectively. (3) Sole maize: the intrarow and interrow spacing of maize plants was 40 and 50 cm, respectively. The row spacings and spacing of cassava remained the same, and the row spacings and spacing of maize remained the same. Therefore, cassava/maize intercropping is equivalent to the superposition of cassava monoculture and maize monoculture.

**Figure 1:**
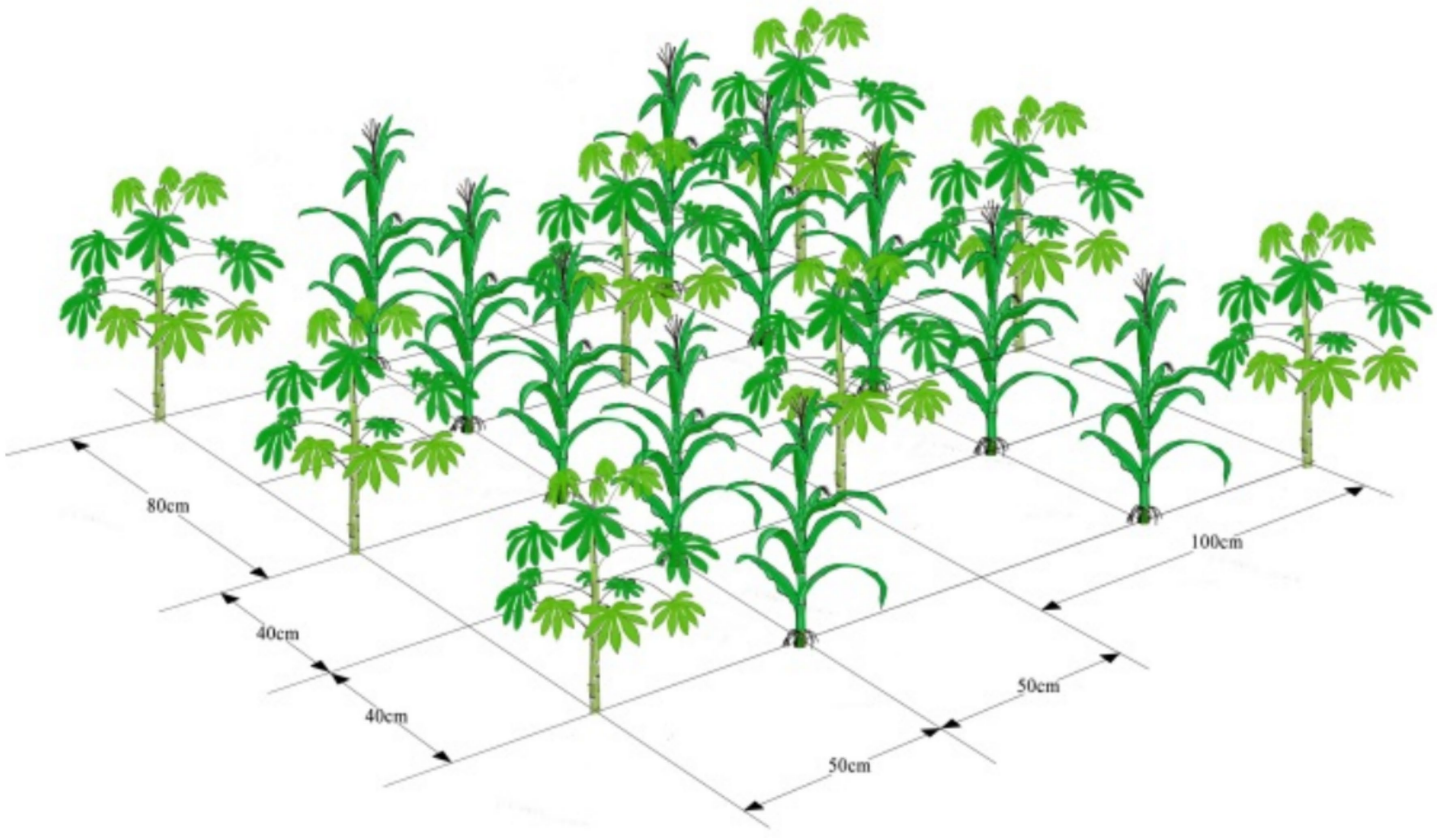
Schematic diagram of planting pattern.

The experiments were laid out using a randomized design with three replicates. Each experimental plot was 32 m^2^ (4 m × 8 m), including four-strip cassava or eight-strip maize or four-strip cassava and eight-strip maize (Figure 1). Maize was sown on 3 March 2021 and harvested on 6 July 2021. Cassava was sown on 3 March 2021 and harvested on 21 December 2021. The plant densities of cassava and maize for both sole crops and intercrops were 125 and 500 plants 100 m^−2^, respectively. The total fertilizer applied twice was 1,500 kg per hectare, and the fertilizer nitrogen, phosphorus and potassium content was 15-15-15. The weeding method was one herbicide combined with artificial weeding.

### 2.2. Soil properties

Soil samples were collected on 11 May 2021 and 70 days after sowing, during the cassava-maize symbiosis period and maize flourishing period. The non-rhizosphere soil was collected around cassava or maize roots, and the rhizosphere soil was collected within 2 mm of the root. At each plot, five to seven soil samples were selected by using an “S”-shaped sampling pattern. Visible roots, organic residues and rock fragments were removed from the soil samples, and the samples were passed through a 2-mm sieve and divided into two subsamples. One subsample was kept fresh by storing it in the refrigerator at −80°C, and one subsample was air dried. The −80°C subsamples were incubated for microbial determination and the amount of microbial carbon nitrogen and phosphorus. The air-dried subsamples were used for the analysis of soil nutrients, such as pH, soil organic matter (SOM), total nitrogen (TN), alkali-hydrolysable nitrogen (Alkali-N), available phosphorus (AP), and available potassium (AK). The concentration of soil nutrients was measured following the protocols described below. The soil pH was determined at a 1:5 solid to liquid ratio (w to v) using deionized water (METTLER FE28). The SOM content was measured using vitriol acid-potassium dichromate oxidation (Nelson, 1996). The TN was determined using the Kjeldahl method and a distillation apparatus (Chen et al., 2021). Alkali-N was measured by the diffusion-absorption method (Roberts et al., 2011). AP was quantified by colorimetric analysis following the extraction of soil with HCl-NH_4_F (Capece et al., 1999). AK was measured by a flame photometer after CH_3_COONH_4_ extraction (Heinen Brown et al., 1999). Microbial biomass C, N and P were measured by the chloroform fumigation-extraction method (Wang et al., 2022). We calculated the microbial biomass C, N, and P as the differences between the fumigated and unfumigated soils. The calculated microbial biomass C, N, and P were divided by 0.45, 0.54, and 0.40, respectively.

### 2.3. Biological analyses

#### 2.3.1. DNA extraction

Total genomic DNA from the soil samples was extracted using the CTAB method. The DNA concentration and purity were determined on 1% agarose gels. After quantification, DNA was diluted to 1 ng μl^−1^ with sterile water.

#### 2.3.2. PCR amplification and PCR product purification

The V4 region of the 16S rRNA genes of bacteria was amplified using the forward primer 515F (GTGCCAGCMGCCGCGGTAA) and reverse primer 806R (GGACTACHVGGGTWTCTAAT) with barcodes. The ITS region of fungal genes was amplified using the forward primer ITS1F (CTTGGTCATTTAGAGGAAGTAA) and reverse primer ITS2R (GCTGCGTTCTTCATCGATGC). PCR was carried out in a 30 μl volume: 15 μl of Phusion® High-Fidelity PCR Master Mix (New England Biolabs), 0.2 μM of both forward and reverse primers, and 10 ng DNA. PCR cycles were completed in a BIO-RAD T100™ Thermal Cycler (USA) at 98°C for 1 min, followed by 30 cycles of denaturation at 98°C for 10 s, annealing at 50°C for 30 s, elongation at 72°C for 30 s, and finally 72°C for 5 min. PCR products were purified by electrophoresis on a 2% agarose gel. Then, the purified PCR products were quantified using a Qubit 2.0 Fluorometer (Thermo Scientific). Equivalent PCR products of different samples were mixed together and purified with a GeneJET Gel Extraction Kit (Thermo Scientific).

#### 2.3.3. Library preparation and sequencing

Sequencing libraries were generated using an Illumina TruSeq DNA PCR-Free Library Preparation Kit (Illumina, USA). The library was qualified using a Qubit 2.0 Fluorometer (Thermo Scientific) and Agilent Bioanalyzer 2100 system. Finally, the library was sequenced on an Illumina HiSeq 2500 platform.

### 2.4. Data analysis

One-way analysis of variance (ANOVA), t test and non-parametric tests were performed to determine the difference between treatments, and the significance was set at *p* < 0.05. All statistical analyses were carried out using SPSS (version 22.0). Alpha-diversity (Shannon, Simpson, and Chao1 indices), Comparison of bacterial community composition between rhizosphere soil and non-rhizosphere soil in different cropping systems was performed through the analysis of the β diversity of the soil microbial community. Principal coordinate analysis (PCoA), redundancy analysis (RDA)/canonical correlation analysis (CCA), variation partitioning analysis (VPA), correlation analysis and figures were performed using the free online Majorbio Cloud Platform.^1^

## 3. Results

### 3.1. Effects of intercropping on soil nutrients

Compared with the initial soil, planting crops reduced the soil pH. The pH of both rhizosphere and non-rhizosphere soil was lower than that of the original soil. The rhizosphere soil pH was lower than that of the non-rhizosphere soil. Cassava and maize relay intercropping can slow the decrease in soil pH. The decrease in soil pH in the cassava and corn monocropping soil was larger, while the decrease in soil pH in the cassava and corn intercropping soil was smaller. Planting increased soil organic matter and available potassium in rhizosphere and non-rhizosphere soil. Rhizosphere soil has a higher content of organic matter and available potassium than non-rhizosphere soil. The increase in non-rhizosphere soil organic matter in monoculture cassava was the largest, followed by maize, and the increase in non-rhizosphere soil organic matter in intercropped cassava and maize was the smallest. The available K content of maize non-rhizosphere soil was higher than that of cassava, and the available K content of intercropped maize non-rhizosphere soil was significantly higher than that of other treatments.

### 3.2. Effects of relay intercropping on bacterial community diversity and community structure

**Figure 2:**
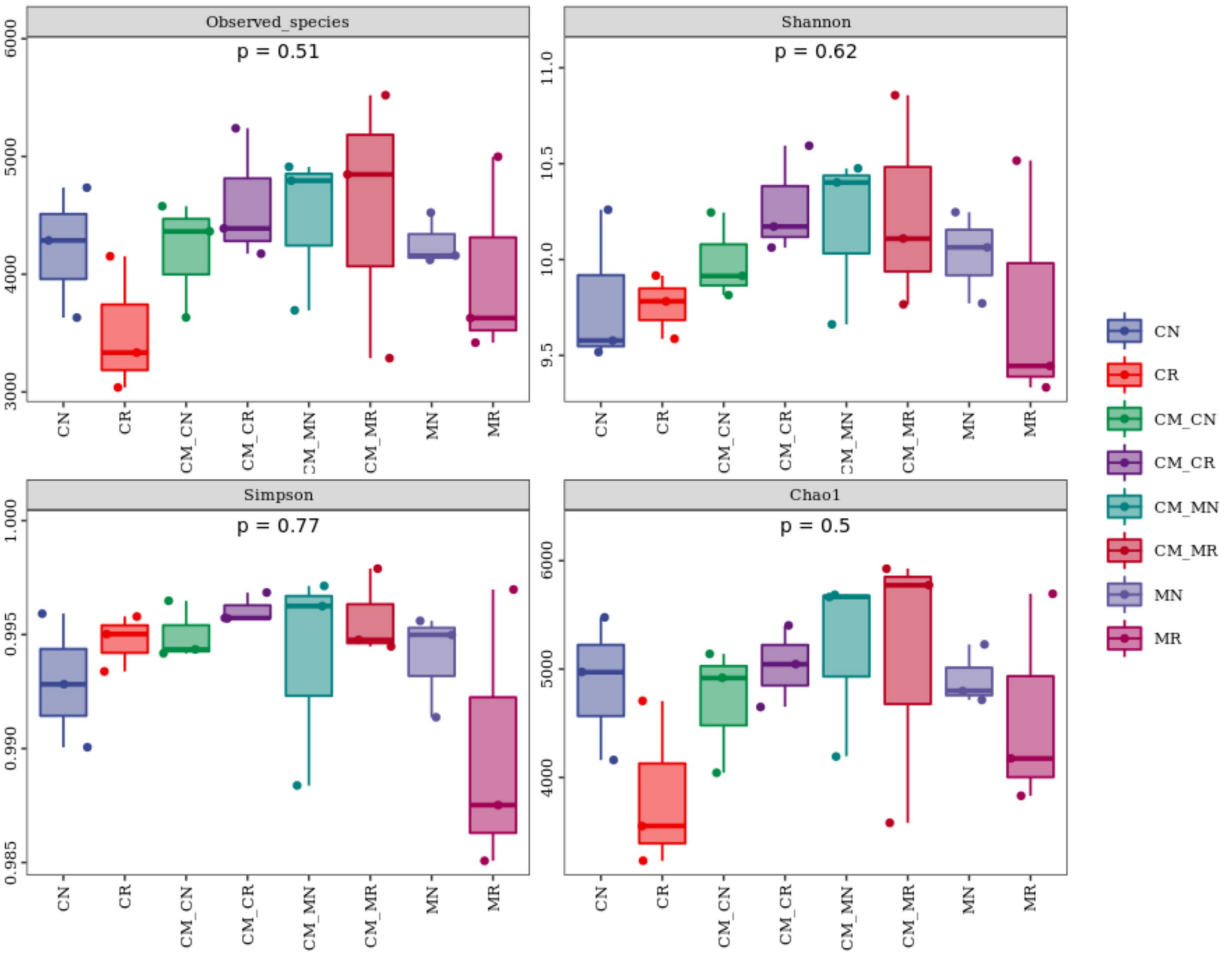
Alpha diversity of the bacterial community in the maize/cassava intercropping systems (*n* = 24, CN: cassava monoculture non-rhizosphere soil; CR: cassava monoculture rhizosphere soil; CM_CN, cassava non-rhizosphere soil in the maize/cassava intercropping system; CM_CR: cassava rhizosphere soil in the maize/cassava intercropping system; CM_MN: maize non-rhizosphere soil in the maize/cassava intercropping system; CM_MR: maize rhizosphere soil in the maize/cassava intercropping system; MN: maize monoculture non-rhizosphere soil, MR: maize monoculture rhizosphere soil).

After filtering, 203, 6,588 reads were obtained, and 52,171 operational taxonomic units (OTUs) were identified with 97% similarity for the bacterial community. The alpha diversity (Observed species, Shannon, Simpson, and Chao1) of the planting patterns was not significantly different (Figure 2). However, observed_species and the Shannon index showed a non-significantly higher trend in the rhizosphere soil than in the non-rhizosphere soil in the crop intercropping system, while that in monoculture system was not significantly lower than that in non-rhizosphere soil.

**Figure 3:**
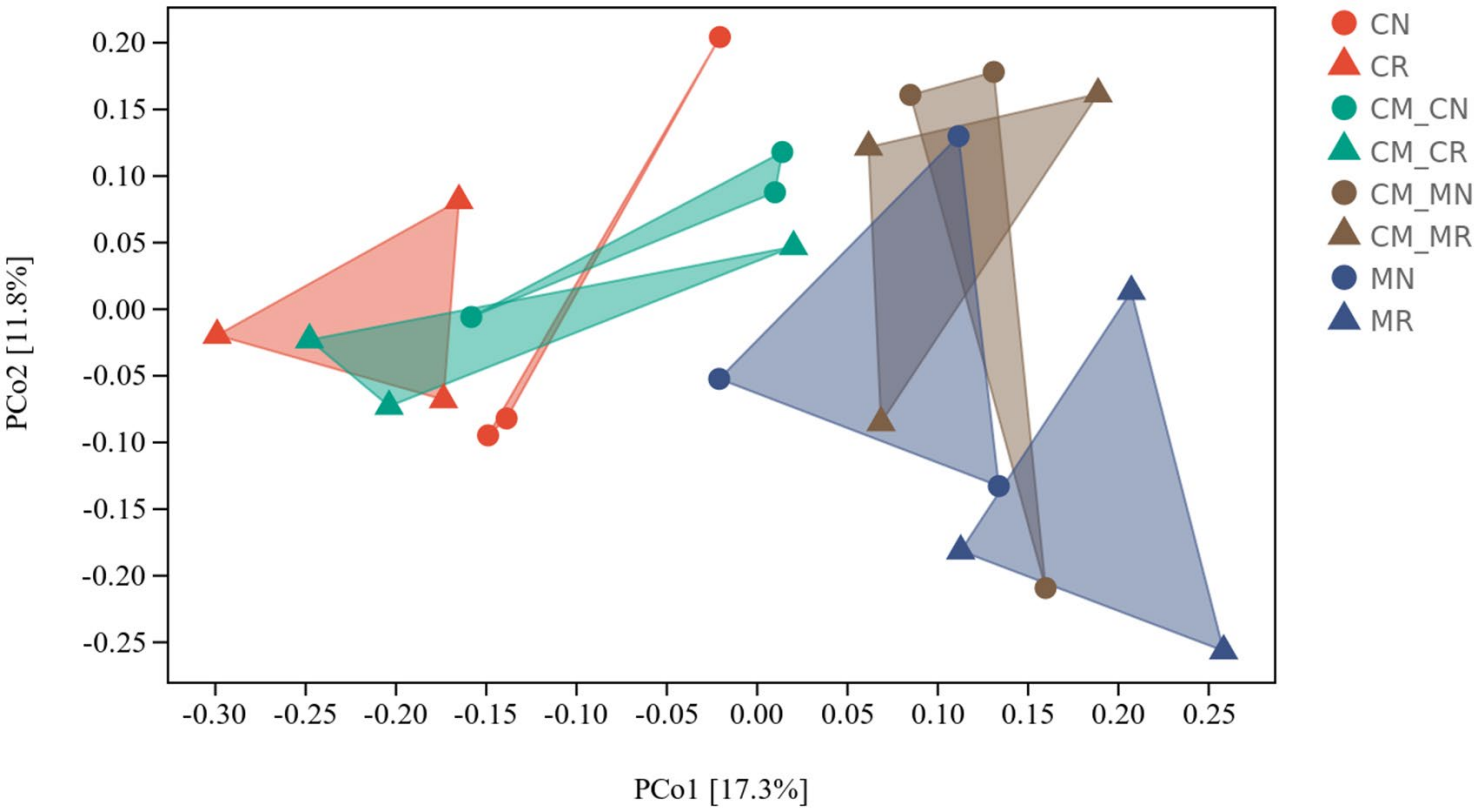
Principal coordinate analysis (PCoA) of bacterial distributions in different intercropping systems. The PCoA plot displays group centroids and dispersions (*n* = 24, CN, cassava monoculture non-rhizosphere soil; CR, cassava monoculture rhizosphere soil; CM_CN, cassava non-rhizosphere soil in the maize/cassava intercropping system; CM_CR, cassava rhizosphere soil in the maize/cassava intercropping system; CM_MN, maize non-rhizosphere soil in the maize/cassava intercropping system; CM_MR, maize rhizosphere soil in the maize/cassava intercropping system; MN, maize monoculture non-rhizosphere soil; MR, maize monoculture rhizosphere soil).

Principal coordinate analysis (PCoA) was used to study the similarity or difference in sample community composition. Bacterial community structures were distinctly grouped by planting pattern on a PCoA plot (Figure 3). In addition to the CM_M group, microbial structures were distinctly grouped by rhizosphere and non-rhizosphere soils. The rhizosphere soil and non-rhizosphere soil samples of different planting patterns were clearly grouped.

### 3.3. Effects of intercropping on soil bacterial composition

**Figure 4:**
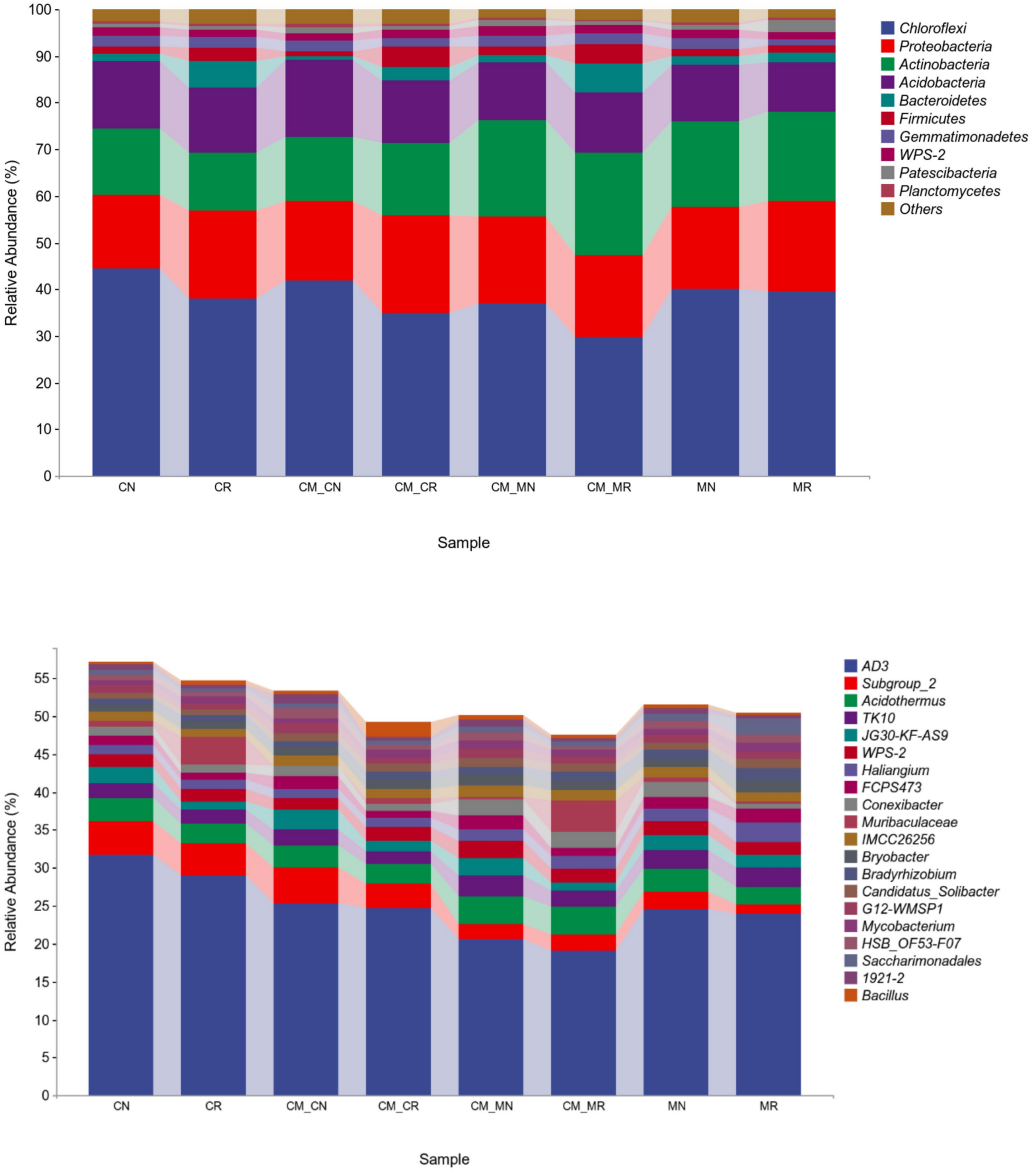
Relative abundance at the phylum (top 10, top) and genus (top 20, bottom) levels of the soil bacterial community of intercropping systems. The bar plot represents the average of samples for each taxon in each cropping system (*n* = 24).

The relative abundance of bacteria at the phylum level and genus level for different planting patterns is shown in Figure 4. Chloroflexi was the most abundant phylum (44%), followed by Proteobacteria (15%), and then Actinobacteria (14%). The relative abundance of Chloroflexi, the dominant phylum, was higher in non-rhizosphere soils than in the corresponding rhizosphere soils. Proteobacteria showed the opposite trend to Chloroflexi.

The most abundant genera in the different cropping systems were *AD3*, *Subgroup_2*, *Acidothermus*, and *TK10*. The relative abundance of *AD3* in each treatment, as the main genus in our experiments, accounted for a large proportion, and the lowest proportion was more than 19%. In different monocropping/intercropping treatments, the order of relative abundance of *AD3* was CR > MR > CM_CR > CM_MR. The relative abundance of *Subgroup_2* showed an increasing trend in cassava crops, and this increase was independent of intercropping.

**Figure 5:**
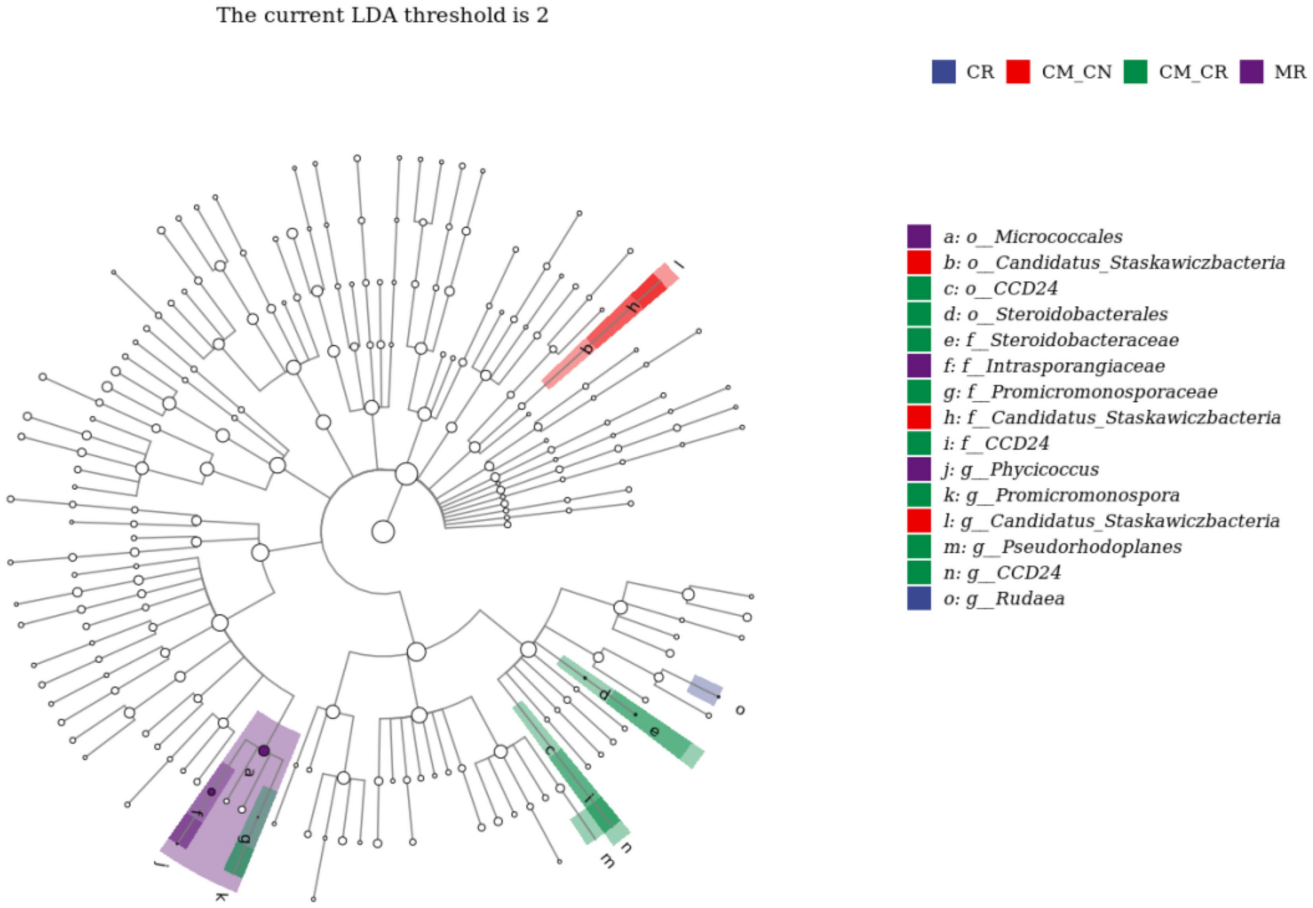
Discriminant analysis of taxa enrichment in each cropping system.

Linear discriminant analysis effect size (LEfSe) revealed one genus in cassava non-rhizosphere soil in the maize/cassava relay intercropping system (CM_CN), *Candidatus_Staskawiczbacteria*, and 3 genera in cassava rhizosphere soil in the maize/cassava relay intercropping system (CM_CR), *Promicromonospora*, *CCD24*, and *Pseudorhodoplanes*. It further revealed one genus in cassava monoculture rhizosphere soil (CR), *Rudaea* (Figure 5), and one genus in maize monoculture rhizosphere soil (MR), *Phycicoccus*.

### 3.4. Relationships between soil properties and the bacterial community

**Figure 6:**
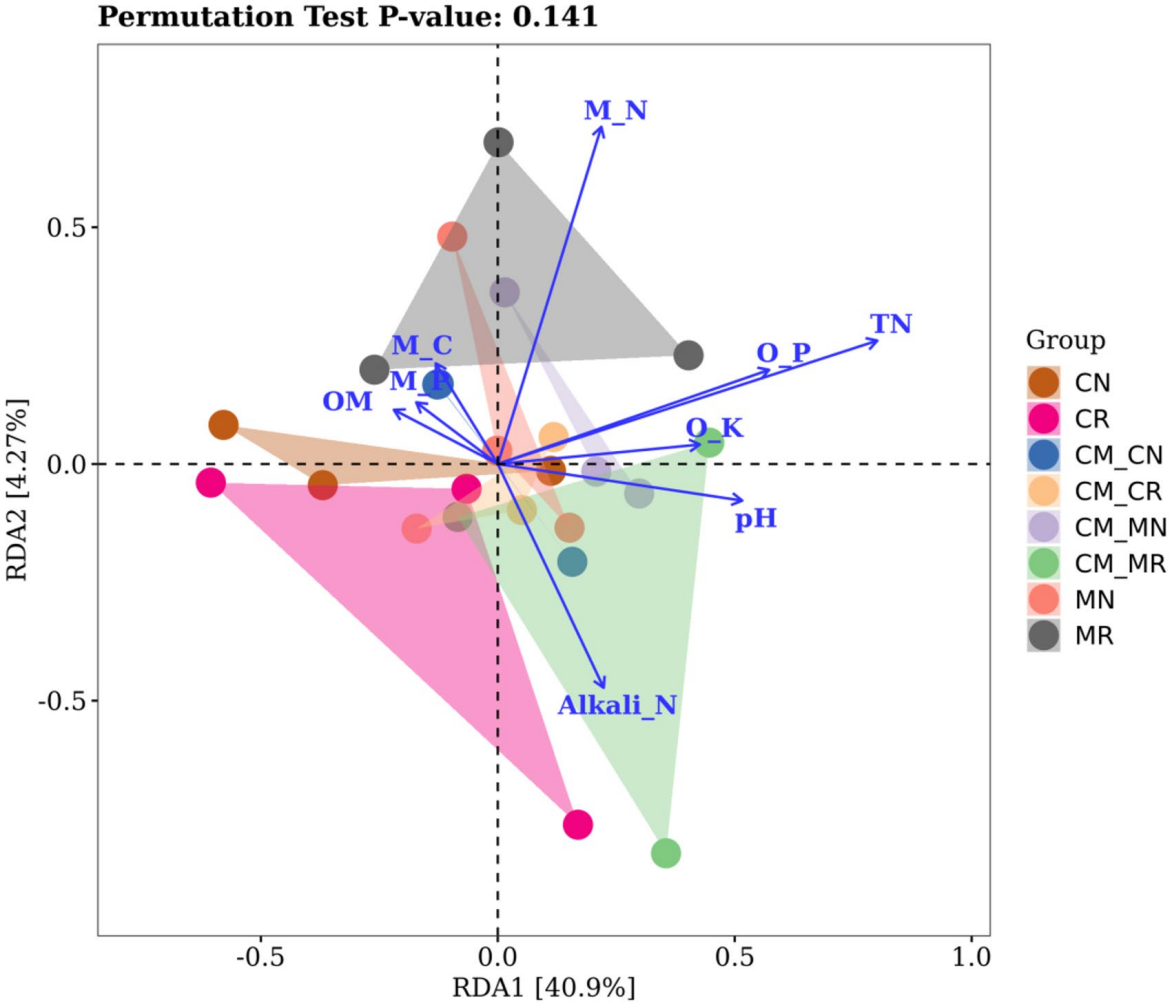
Redundancy analysis (RDA) of MiSeq data (symbols) and environmental factors (arrows; *n* = 24).

Redundancy analysis (RDA) can explain the relationship between environmental factors and the soil microbial community well. As shown in Figure 6, in the analysis of the bacterial community, the total interpretation rate of the RDA1 and RDA2 axes reached 45.1%. The analysis of the bacteria at the genus level showed that under the constraints of soil nutrient factors. Under the constraints of these environmental factors, the distinction between rhizosphere soil and non-rhizosphere soil is more evident. Bacterial communities appear to be closely correlated with TN, microbial nitrogen (M_N), available phosphorus (AP) and pH.

## 4. Discussion

Increased crop yield as well as improved nutrient use efficiency are considered advantages of intercropping as an eco-friendly system (Ben-chuan et al., 2022; Cuartero et al., 2022). In our study, we found that relay intercropping patterns could effectively change soil physical properties and nutrient content.

Compared with the original soil, the organic matter of the non-rhizosphere and rhizosphere in each treatment was significantly improved, and the organic matter content of monocropping was higher. For non-rhizosphere soil organic matter content, it increases in cassava was higher than that in maize. The non-rhizosphere soil organic matter of cassava decreased by 18.4% in intercropping compared with that of monoculture, while that of maize decreased by 8.7% (Table 1). Giagnoni et al. (2022) indicate that planting crops reduces soil organic matter. The reason our results was different with Giagnoni et al. (2022) were probably that sampling sites. The soil samples in our study were non-rhizosphere and rhizosphere soil, which near the roots. Elevation of organic carbon in non-rhizosphere and rhizosphere soils is largely caused by inputs of nutrients that are mainly derived from rhizodeposition. On the one hand, root exudates can increase soil organic matter (Herre et al., 2022). On the other hand, roots can absorb organic matter (Jones and Darrah, 1996).

**Table 1:** Soil properties and nutrients.

	pH	Soil organic matter g/kg	Total nitrogen %	Alkali:nitrogen mg/kg	Available phosphorus mg/kg	Available potassium mg/kg	Microbial carbon mg/kg	Microbial nitrogen mg/kg	Microbial phosphorus mg/kg
CN	3.87 ± 0.05 c	49.94 ± 4.62 a	1.07 ± 0.05 b	69.8 ± 3.22 a	33.0 ± 6.08 a	83.0 ± 4.6 cd	140 ± 36.4 a	17.4 ± 1.8 c	0.118 ± 0.023 e
CR	3.80 ± 0.09 c	50.07 ± 4.15 a	1.09 ± 0.06 b	72.9 ± 3.67 a	44.3 ± 3.64 a	115 ± 4.9 cd	75.6 ± 13.6 cde	18.2 ± 0.7 c	0.128 ± 0.035 e
CM_CN	4.02 ± 0.16 ab	40.72 ± 3.41 c	1.09 ± 0.06 b	70.8 ± 2.91 a	31.8 ± 5.68 a	74.3 ± 5.8 d	51.3 ± 9.8 e	20.9 ± 6.9 c	0.328 ± 0.209 de
CM_CR	3.97 ± 0.03 b	46.46 ± 1.68 ab	1.15 ± 0.04 ab	74.5 ± 1.95 a	35.1 ± 0.76 a	100 ± 9.9 cd	82.6 ± 8.0 cd	34.8 ± 2.6 b	0.220 ± 0.100 e
CM_MN	4.11 ± 0.05 a	40.31 ± 1.43 c	1.16 ± 0.06 ab	70.2 ± 3.67 a	44.7 ± 2.70 a	140 ± 8.1 bc	60.4 ± 4.7 de	37.6 ± 4.8 b	0.760 ± 0.105 bc
CM_MR	3.99 ± 0.08 b	42.10 ± 0.99 bc	1.22 ± 0.03 a	74.8 ± 3.88 a	40.2 ± 3.61 a	244 ± 34.4 a	145 ± 16.3 a	46.0 ± 5.6 a	0.942 ± 0.084 b
MN	3.93 ± 0.13 bc	44.16 ± 2.24 bc	1.12 ± 0.06 ab	70.6 ± 2.64 a	39.6 ± 5.66 a	105 ± 9.1 cd	125.1 ± 11.4 ab	24.7 ± 1.7 c	0.543 ± 0.121 cd
MR	3.90 ± 0.08 c	49.28 ± 1.47 a	1.18 ± 0.04 ab	74.9 ± 3.67 a	45.1 ± 6.33 a	197 ± 29.7 ab	96.9 ± 11.3 bc	46.2 ± 5.9 a	1.052 ± 0.343 a

ANOVA, Duncan’s test; the letters indicate *p* < 0.05; *n* = 3; CN, cassava monoculture non-rhizosphere soil; CR, cassava monoculture rhizosphere soil; CM_CN, cassava non-rhizosphere soil in the maize/cassava intercropping system; CM_CR, cassava rhizosphere soil in the maize/cassava intercropping system; CM_MN, maize non-rhizosphere soil in the maize/cassava intercropping system; CM_MR, maize rhizosphere soil in the maize/cassava intercropping system; MN, maize monoculture non-rhizosphere soil; MR: maize monoculture rhizosphere soil.

As for the impact of intercropping on organic matter content, Wang et al. (2021), pointed out that intercropping increased the concentration of soluble organic carbon in organic matter, making its chemical composition more complex (Wang et al., 2021), while our results showed that the content of organic matter under relay intercropping was lower than that under monocropping. The reason may be that the biomass per plant of cassava and maize was lower under relay intercropping (Olasantan et al., 1996), which resulted in a smaller amount of root exudates and a smaller increase in soil organic matter. Intercropping increases the number of plants planted, make more soil become non-rhizosphere and rhizosphere soil.

Planting crops can acidify soil and reduce soil pH (Giagnoni et al., 2022). Our results showed that planting cassava and maize decreased soil pH, but intercropping slowed the decreasing trend, and the pH of rhizosphere soil was lower than that of non-rhizosphere soil. The pH in the plant rhizosphere is mainly controlled by the release of low-molecular-weight organic acid and CO_2_ in the soil solution and by the H^+^ and OH^−^ ions released by roots to balance the charge upon uptake of anionic nutrients (Hinsinger et al., 2009). This can explain the observed pH reduction in rhizosphere soil, and the change in soil pH has a significant impact on the available state of microorganisms and soil nutrients.

Some plant root exudates can activate mineral potassium in soil, but no reports have been found on this function of maize. Our results showed that the content of available potassium in maize rhizosphere soil increased more than that in cassava rhizosphere soil. Root exudates improve soil organic matter due to the organic acids contained in them, which promote the mineralization of soil organic matter and dissolve the potassium contained in the soil parent material to make it available. Small molecular organic acids secreted by roots can improve soil organic matter, reduce soil pH, promote the mineralization of soil organic matter, and promote the conversion of potassium in soil into an available state. The change trend of the soil available potassium content was closely related to crop growth. The potassium absorption capacity of maize seedlings was weak, and the requirement of potassium was low. The soil available potassium content in the rhizosphere and non-rhizosphere could maintain a high level (Han et al., 2017).

Some studies have noted that the release of K is positively correlated with its absorption (Taiwo et al., 2018). In our study, we found that the soil near the corn roots already has a large amount of available potassium for the crop to absorb before the plant begins to absorb large amounts of potassium. Since activated K from maize can be absorbed by intercropped cassava, this also explains the phenomenon of low available K content in cassava and intercropped cassava soil in our experiment. Relay intercropping not only made rational use of soil in time and space but also promoted the efficient use of soil nutrients due to the changes in soil nutrient forms caused by different crops. Soil organic matter, pH value and available potassium were significantly affected by intercropping. Based on the above analysis, we speculated that intercropping was caused by the effect of crop roots.

In our study, we found that the bacterial community structure differed significantly between relay intercropping and monocropping systems and that its effect was mainly concentrated on the microbes in the rhizosphere soil. Because exudates released from plant roots are the main source of nutrients for microorganisms to drive their population density and activity, rhizosphere soil has become a trending topic in the study of microbial-plant interactions (Gong et al., 2019; Bian et al., 2021). These differences also suggest that intercropping may affect the abundance of some microbial populations in the soil but not population diversity. *AD3*, which is an absolutely dominant genus, has many functions. Xie et al. (2021) found that *AD3* has good resistance to Cd contamination and ultimately changes the forms of C, N and P (Li et al., 2020). The decrease in the abundance of *AD3* may be related to the decrease in the bioavailability of Cd, which is related to the change in pH caused by intercropping. The changes in organic matter and total nitrogen are also related to the participation of *AD3*, so intercropping may be related to the bioavailability of Cd. Based on the function of the differential bacteria screened by LEfSe, the relative abundance of *Phycicoccus* in MR was related to phenanthrene pollution, and the relative abundance of *Phycicoccus* was reduced in CM_MR. As an indicator of phenylalanine contamination, the decline in the relative abundance of *Phycicoccus* suggested that intercropping may also have potential in the elimination of organic pollutants. While the relative abundance *Rudaea* (a pathogenic species) was higher in cassava monocropping treatments, its relative abundance was correlated with that of other pathogenic bacteria (Li et al., 2021). In our study, intercropping non-significantly increased the Shannon and Chao1 indexes, indicating that the community diversity changed, resulting in a lower relative abundance of pathogenic bacteria. The relative abundance of the genera *Promicromonospora* and *Pseudorhodoplanes* in CM_CR increased with atrazine application (Liu et al., 2020). Atrazine is a commonly used herbicide in maize fields. The increased relative abundance of microorganisms in these two genera, which are biomarkers of atrazine, also suggests that more attention may need to be paid to pesticide residues in intercropping systems.

## 5. Conclusion

Compared with monocropping, Soil organic matter, pH value and available potassium were significantly affected by relay intercropping. We speculated that intercropping was caused by the effect of crop roots. The bacterial community was characterized by a higher abundance of the phyla Chloroflexi, Proteobacteria, and Actinobacteria and of the genus *AD3*, which are related to nutrient cycling and the decrease in the bioavailability of Cd. At the same time, relay intercropping reduced the relative abundance of pathogenic bacteria and biomarkers of organic pollution. The effect of intercropping did not significantly affect the α-diversity of bacteria in a short period of time, but the change in β-diversity was significant. At the same time, environmental factors such as TN, M_N, AP, and pH were identified, which are considered to have a significant impact on the diversity of soil bacteria.

## Data availability statement

The datasets presented in this study can be found in online repositories. The names of the repository/repositories and accession number(s) can be found at: Sequence Read Archive, accession number: SRP410378.

## Author contributions

CH: resources and writing – original draft. BZ: conceptualization, writing – reviewing, and funding acquisition. HW: methodology, investigation, and funding acquisition. YW: writing – reviewing. JH: conceptualization, project administration, and funding acquisition. All authors contributed to the article and approved the submitted version.

## Funding

This work was supported by the National Key R & D Program of China (2020YFD1000603), China Agriculture Research System (CARS-11-HNHJ), Project funded by China Postdoctoral Science Foundation (2021M692144) and Shanghai Post-doctoral Excellence Program (2021304).

## Conflict of interest

The authors declare that the research was conducted in the absence of any commercial or financial relationships that could be construed as a potential conflict of interest.

## Publisher’s note

All claims expressed in this article are solely those of the authors and do not necessarily represent those of their affiliated organizations, or those of the publisher, the editors and the reviewers. Any product that may be evaluated in this article, or claim that may be made by its manufacturer, is not guaranteed or endorsed by the publisher.

## Footnotes

^1^ www.majorbio.com
